# *FTO* Common Obesity SNPs Interact with Actionable Environmental Factors: Physical Activity, Sugar-Sweetened Beverages and Wine Consumption

**DOI:** 10.3390/nu14194202

**Published:** 2022-10-09

**Authors:** Danyel Chermon, Ruth Birk

**Affiliations:** Nutrition Department, Health Science Faculty, Ariel University, Ariel 40700, Israel

**Keywords:** FTO, body mass index, single nucleotide polymorphisms, obesity, physical activity

## Abstract

Genetic background is estimated to play >50% in common obesity etiology. *FTO* single nucleotide polymorphisms (SNPs) are strongly associated with BMI, typically in European cohorts. We investigated the interaction of common *FTO* SNPs with actionable environmental factors, namely physical activity, sugar-sweetened beverages (SSB) and wine consumption, and verified *FTO* common SNPs predisposition to obesity in the Israeli population. Adults’ (>18 years old, *n* = 1720) *FTO* common SNPs data and lifestyle and nutrition habits questionnaires were analyzed using binary logistic regression models, adjusted for confounding variables (age, sex) assuming dominant, recessive and additive genetic models. Eighteen *FTO* SNPs were associated with significant increased obesity risk and interacted with physical activity (*p* < 0.001), wine consumption (*p* < 0.014) and SSB consumption (*p* < 0.01). Inactive rs9939609 risk-allele carriers had significantly higher obesity risk compared to their active counterparts (OR = 2.54, 95% CI 1.91–3.39 and OR = 3.77, 95% CI 2.47–5.75; *p* < 0.001 with 3.1 and 3.5 BMI increment for heterozygotes and homozygotes, respectively). SSB consumption (≥1 serving/day) significantly raised obesity risk and wine consumption (1–3 drinks/weekly) significantly lowered obesity risk for rs9939609 risk-allele carriers (OR = 1.54, 95% CI 1.05–2.27; *p* = 0.028 and OR = 0.61, 95% CI 0.47–0.79; *p* < 0.001, respectively). Our findings demonstrate that actionable lifestyle factors modify the common *FTO* obesity risk in predisposed carriers, and they have personal and public health implications.

## 1. Introduction

The etiology of common obesity involves both environmental and genetic factors. It has been estimated that body mass index (BMI) heritability is high, ranging between 40% and 70% [1]. Common polygenic obesity is attributed to several hundreds of genetic polymorphisms, each with a relatively small effect [2]. The genetic predisposition to common obesity is phenotypically expressed with exposure to an obesogenic and sedentary environment. An obesogenic environment, shared by developed and developing countries worldwide [3], is characterized by calorie-dense food, pollutants, speed eating, large and increasing food portion size, high consumption of sugar-sweetened beverages (SSBs), and excessive intake of simple carbohydrates and sugars. Additionally, sedentary lifestyle, characterized by elevation in screen-related immobility, insufficient sleep and lack of adequate physical activity, contribute to the sharp elevation in obesity rates [4,5].

The fat mass and obesity associated (*FTO*) gene, located on chromosome 16, was identified through mutant mouse studies [6]. *FTO* is expressed in many tissues, both in peripheral and central-feeding brain regions such as arcuate, paraventricular and ventromedial nuclei [7]. The *FTO* gene encodes an alpha-ketoglutarate-dependent dioxygenase that through the methylation of DNA/RNA regulates transcription and translation [8]. Although *FTO’s* role in the development of obesity is not entirely clear, common polymorphisms in the *FTO* gene are strongly associated with obesity [9], BMI [10,11], increased energy and dietary fat intake [12,13], increased appetite and reduced satiety [14] as well as poor food choices and eating habits [15]. In fact, common *FTO* gene polymorphisms have the largest effect known on BMI and obesity risk [16] across ancestries [17,18,19], among adults, children and adolescents [10,11,16]. Of the 21 identified *FTO* single nucleotide polymorphisms (SNPs), 18 have shown strong association and have been replicated, mostly in European descent cohorts [20] with *FTO* SNP rs9939609 being the most studied [21]. It was suggested that *FTO* SNPs’ predisposition to obesity is race-specific, as the relationships between rs9939609 and obesity is inconsistent [22,23]. Previous studies of the interaction of *FTO* common SNPs with lifestyle factors, focusing mainly on rs9939609, found that obesity predisposition due to *FTO* SNPs can be modified by physical activity, attenuating increase in BMI [24,25]. Evidence for interaction was also found with SSBs in relation to BMI [26] and for dietary intake. However, reports in this regard are controversial and need further investigation [27,28].

Given that genetic predisposition to obesity is inherited and thus unchangeable, identifying actionable environmental factors that interact with this disposition is essential for treatment. Furthermore, different common *FTO* SNPs show variable association with obesity in different populations, thus challenging the inclusion of obesity risk estimates in other world populations. This study aims to investigate the interaction of common *FTO* SNPs with actionable environmental factors, including physical activity and drinking habits (SSB and wine consumption), in determining and modifying the risk of obesity through studies of the Israeli population.

## 2. Materials and Methods

### 2.1. Participants

Healthy adults (*n* = 1972), 1377 (79% females), mean aged 55.22 ± 14.36 data (21 December 2021–26 July 2022) were studied. The analysis in this work is based on information listed in Israeli registry database (#700068969) of Lev Hai Genetics LTD—MyGenes. Non-identifiable genetic data were used. The study was approved by the ethical committee of Ariel university (#AU-HEA-RB-20220214). Participants under the age of 18 years, having a genetic disease, diabetes or missing anthropometric data were excluded from the study (*n* = 40).

Data on physical activity and drinking habits were obtained as a part of an online lifestyle questionnaire filled by 1796 (90%) of study participants. Questions regarding physical activity habits included: “Are you physically active” (possible answers: yes; no); “How many days a week do you engage in physical activity” (answer: number of days participant is physically activity); “What is your physical activity duration” (possible answers: up to 30 min; at least 30 min; more than 60 min); Physically activity was defined as engaging ≥ 30 min/day in physical activity. Questions regarding drinking habits included; “How many cups of sugar-sweetened beverages do you drink on a daily basis?” (possible answers: none; 1–3 daily cups; more than 3 daily cups); “How many drinks of wine do you drink on a weekly basis” (possible answers: none; 1–3 weekly drinks; more than 3 weekly drinks)”. SSBs consumption was defined as having at least 1 daily serving equals 12 fluid ounces of SSB and regular wine consumption was defined as having 1–3 weekly drinks of wine; one drink equals 5 fluid ounces of wine.

Weight and height were self-reported. Height was reported in centimeters and weight in kilograms. BMI was calculated as the ratio of weight/(height)^2^ (kg/m^2^). Subjects were classified as obese (BMI ≥ 30) or not obese (BMI < 30) according to BMI cutoff points [29].

### 2.2. SNP Selection and Hardy–Weinberg Equilibrium (HWE)

SNPs that were previously shown to be significantly associated with obesity were selected. These SNPs were prioritized based on their minor allele frequency (MAF) (>0.01) in at least two GWAS populations [10,11,16,17,20,30,31,32,33,34] and based on the validated catalog of published genome-wide association studies [35]. The SNPs included: rs9939609, rs1421085, rs8050136, rs8051591, rs3751812, rs9935401, rs11075989, rs9923233, rs9936385, rs17817964, rs8043757, rs1121980, rs17817449, rs62033400, rs7202116, rs7193144, rs11075990, rs6499640, rs13333228, rs1558902 and rs9302652. Hardy–Weinberg equilibrium (HWE) was tested for each SNP using a 1 degree of freedom χ^2^-test, and all studied common *FTO* SNPs were in HWE.

### 2.3. Statistical Analyses

An a priori power analysis was conducted using G*Power 3.1.9.7 software [36] in order to determine the study sample size. The analysis revealed that using a sample of 80 participants across two groups was sufficient to observe the association between SNP and obesity (α = 0.05, with an odds ratio (OR) = 2 and a power of 0.80). We used a chi-square statistical test to compare dichotomic characteristic variables between obese and non- obese participants and Mann–Whitney for independent groups for continuous variables that were not normally distributed. Continuous variables are presented as mean values ± standard deviation. Binary logistic regression was performed to test the hypothesis of association between polymorphisms as predictors and their effect on the relative likelihood of obesity (BMI ≥ 30) after adjustment for potential confounders (age, gender). Logistic regression with interaction was used to determine the OR of gene–environment interaction with obesity. We determined whether the association of genotype with obesity risk was modified by physical activity and drinking habits by constructing a regression model that included the following independent variables: sex, age, physical activity (yes/no; yes regards for at least 30 min/a day regularly), regular SSBs consumption (≥1 daily serving/not consuming) and regular wine consumption (1–3 weekly drinks/not consuming) × genotype. The presence of an interaction between actionable lifestyle variables (physical activity and drinking habits) and SNPs genotype on obesity risk was assessed by a likelihood ratio test, in which we compared genotypes SNPs model with the interaction term. Statistical analyses were performed using SPSS 27.0 for Windows (SPSS Inc., Chicago, IL, USA).

## 3. Results

### 3.1. Participants Characteristics

This cross-sectional study included 1972 (79% female) Israeli adult participants 55.22 ± 14.36. Population characteristics are shown in Table 1. Men were significantly more obese than women (*p* < 0.001). Risk of obesity for men was 1.5-fold higher compared to women. There was a significant difference in being physically active (≥30 min/day), consuming SSB (≥1 serving/day) and regularly drinking wine (1–3 drinks/week) between participants defined as obese (BMI ≥ 30) and participants defined as non-obese. Specifically, participants defined as obese were significantly less active compared to participants defined as non-obese (*p* < 0.001). Among the obese group, there were significantly less participants who consumed wine and more participants who consumed SSBs (*p* < 0.001 and *p* = 0.002), respectively. Age, height and smoking status did not differ between participants defined as obese and participants defined as non-obese.

### 3.2. FTO SNPs Association and Obesity Risk

We analyzed twenty-one *FTO* SNPs with previously reported association to obesity. Three of the SNPs were found to be non-significant in relation to obesity, and all other eighteen studied SNPs were significantly related to elevated obesity risk with OR ranging from 1.2 to 1.35 and exhibited strong linkage disequilibrium (r^2^ = ~1) corresponding to CEU population. Thus, we focused on *FTO* rs9939609 as a representative of all SNPs. The distribution of *FTO* rs9939609 genotypes and OR for dominant, recessive additive and codominant genetic models adjusted for age and gender are shown in Table 2.

Among all individuals tested for the *FTO* rs9939609 variant, 24.8% were homozygous (AA), 46.9% were heterozygous (TA), and 28.2% were wild type (TT). Follow-up regression analysis of *FTO* SNPs homozygous individuals compared to heterozygotes variants carriers indicated that the additional allele did not contribute to a higher OR had a very weak contribution, indicating that the SNP association to obesity follows a dominant model. Compared to wild type, a 0.65 BMI increment was found for rs9939609 variant heterozygous and 1.2 BMI increment for rs9939609 variant homozygous (*p* < 0.001).

### 3.3. Gene Environment Interactions

#### 3.3.1. Physical Activity Interaction with FTO on Obesity Risk

All eighteen *FTO* SNPs had a significant interaction with physical activity (*p* < 0.001) (Table S1). The frequency of *FTO* rs9939609 genotypes did not differ significantly between physically active and inactive participants (*p* = 0.38). Specifically, the distribution of *FTO* rs9939609 genotypes regarding physical activity was 240 (53.7%) homozygous (AA), 413 (49%) heterozygous (TA), and 255 (50.4%) wild type (TT). Although there was no significant difference in genotype frequency between active and inactive individuals, there was a significant difference in mean weight, BMI and obesity status between *FTO* rs9939609 genotypes (*p* = 0.008, *p* = 0.004 and *p* = 0.013, respectively) (Table 3).

As shown in Table 4, obesity risk was significantly higher among *FTO* rs9939609 risk-allele carriers that were physically inactive as compared to non-risk inactive counterparts, both in the dominant, recessive and codominant models. Obesity risk was higher in inactive individuals homozygous to *FTO* rs9939609 compared to inactive individuals heterozygous to *FTO* rs9939609 (OR = 1.78, 95% CI 1.23–2.57; *p* = 0.002 and OR = 1.4, 95% CI 1–1.93; *p* = 0.04), respectively. There was no significant difference in obesity risk between physically active risk carriers, both heterozygous or homozygous to *FTO* rs9939609 variant, compared to physically active wild types.

We further analyzed effects of gender in relation to obesity risk interaction with physical activity. We found a significant effect of gender on obesity risk interaction with physical activity. Obesity risk for inactive *FTO* rs9939609 homozygous men was significantly higher compared to inactive homozygous women (OR = 3, 95% CI 1.2–7.52; *p* = 0.019 and OR = 1.58, 95% CI 1.05–2.38; *p* = 0.03, respectively).

Table 5 shows the effect of potential lifestyle variables on *FTO* rs9939609 risk-allele carriers and obesity risk. After adjusting for potential confounders (age and gender), inactive individuals *FTO* rs9939609 risk-allele carriers (TA and AA) demonstrated higher obesity risk compared to their physically active counterparts (β = 1.048, *p* < 0.001). Inactive non-risk carriers had 2.29 times higher obesity risk compared to active counterparts. Inactive *FTO* rs9939609 risk-allele homozygotes had 3.77 times higher obesity risk compared to active homozygotes. Inactivity significantly increased risk of obesity even in wild-type individuals (TT carriers).

#### 3.3.2. Wine Consumption Interaction with FTO Polymorphism in Determining Obesity Risk

Wine consumption significantly interacted with all eighteen *FTO* SNPs in determining *FTO* SNPs-related obesity, assuming a dominant model. Regardless of genotype, the regular consumption of one to three weekly drinks of wine reduced obesity risk by 36% (*p* < 0.001) in our sample (not shown in the table).

Weekly wine consumption reduced the risk of obesity by 44% (OR = 0.56) for the wild type (TT) (*p* = 0.009) and by 32% (OR = 0.68) for those who carried at least one *FTO* rs9939609 risk allele (*p* = 0.005) compared to their non-drinking counterparts *(n* = 494 and *n* = 1260, respectively) (Table 5). Thus, the risk of obesity among risk-allele carriers regularly drinking wine was reduced to that of non-risk carriers.

#### 3.3.3. SSBs Consumption Interaction with FTO on Obesity Risk

SSB consumption of ≥1 serving/day significantly increased obesity risk (OR = 1.5, 95% CI 1.1–2.06; *p* = 0.011).

Consuming SSBs significantly interacted with *FTO* rs8050136, rs1421085, rs9939609 and rs1121980 in risk-allele carriers to effect obesity (*p* = 0.013, *p* = 0.015 *p* = 0.049 and *p* = 0.017), respectively.

*FTO* rs9939609 A allele carriers who consumed ≥1 serving of SSB on a daily basis had a 1.5-fold increased risk of obesity compared to A allele carriers who did not consume any SSBs (*p* = 0.028). Interestingly, when stratified to gender, obesity risk was higher but remained significant only for rs9939609 heterozygous women (OR = 2.23, 95% CI 1.33–3.76; *p* = 0.002). The same results were shown for the other three SNP’s (Table 6).

**Figure 1:**
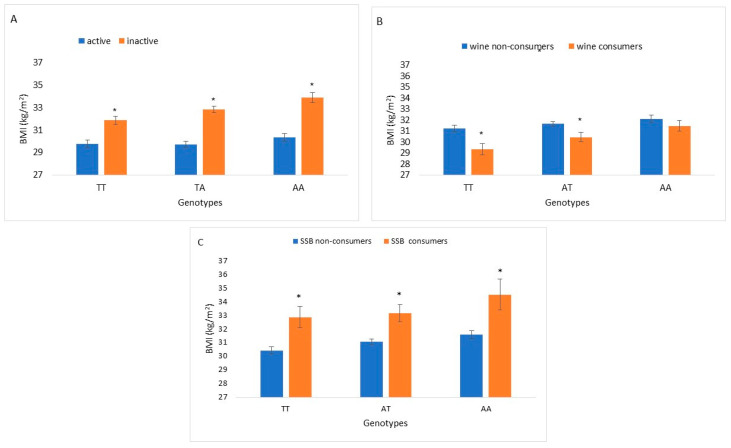
BMI increments of *FTO* rs9939609 genotypes interaction with actionable environmental factors: physical activity (*n* = 1735), wine consumption (*n* = 1754) and SSB consumption (*n* = 1796). (**A**): BMI of physical activity stratified by *FTO* rs9939609 genotypes. Bars indicate BMI levels of active and inactive *FTO* rs9939609 wild type (TT), heterozygous (TA) and homozygous (AA). (**B**): BMI of wine consumption stratified by *FTO* rs9939609 genotypes. Bars indicate BMI levels of participants consuming 1–3 drinks/week and non-wine consumers among *FTO* rs9939609 genotypes. (**C**): BMI of SSBs (≥1 serving/day) consumption stratified by *FTO* rs9939609 genotypes. Bars indicate BMI levels of participants consuming ≥1 serving of SSB/day and non-SSB consumers among *FTO* rs9939609 genotypes. Differences between BMI levels within each genotype group (TT, TA and AA) were tested by Mann–Whitney for 2 independent samples. Asterisks represent statistically significant difference *p* < 0.05.

BMI levels were significantly different between active and inactive participants and between SSB consumers and non-consumers across all FTO rs9939609 genotype groups. Significant differences in BMI levels were found between wine consumers and non-consumers within the wild-type (TT) and heterozygote (TA) genotypes (Figure 1).

## 4. Discussion

We demonstrate that *FTO* common obesity SNPs interact with actionable environmental factors and are associated with obesity in the Israeli population. All 18 studied SNPs were significantly associated with elevated obesity risk and interacted with physical activity and wine consumption as obesity risk modulators. Four SNPs interacted with SSBs consumption to enhance obesity risk.

We show a significant modulating interaction between physical activity and *FTO* rs9939609 on obesity risk. To date, publications regarding *FTO* rs9939609 obesity risk-allele carriers’ interaction with physical activity show inconsistency in different populations [37,38,39,40], stressing the importance of studying this interaction in different populations. In our study, we found no difference in obesity risk between physically active rs9939609 variant carriers and physically active non-carriers. *FTO* rs9939609 risk-allele carriers who were physically active had 25% lower risk for obesity compared to risk-allele carriers who were not physically active, where homozygous risk carriers had a 2.03 BMI increment compared to active counterparts (*p* = 0.004). This increment is higher than previous reports showing 0.44 and 1.95 BMI increment per risk allele in inactive rs9939609 A-allele homozygotes carriers compared with T-allele homozygotes [41,42]. The mechanism underlying the effect of physical activity on *FTO* might be related to *FTO’s* potential role in the regulation of energy homeostasis [8]. Our results suggest that physical activity is an important key factor that might attenuate and prevent the adverse effect of *FTO* SNPs on obesity for susceptible individuals, reinforcing the importance of physical activity as a strategy for the treatment and prevention of obesity in general and for genetically designated individuals in particular. The appropriate activity and duration should be further investigated for the precise protocol for different genetic architecture.

Further investigating actionable interactions between lifestyle factor and *FTO* polymorphisms, we studied the interaction between wine consumption and *FTO* rs9939609 variation on obesity risk. Previous studies mostly investigated interactions between *FTO* variants and alcohol consumption and risk for alcohol dependence but not the risk of obesity [43]. Several studies showed that *FTO* SNPs (including rs8062891, rs1108086, rs1420318, rs12597786 and rs7204609) had significant associations with alcohol dependence [44]. Our study is to the best of our knowledge the first to provide evidence for a possible interaction between wine consumption and *FTO* risk predisposition to obesity. We show that wine consumption (1–3 weekly drinks) reduced the risk of obesity by 32% for rs9939609 A carriers who had at least one risk allele, suggesting that wine has a protective effect against obesity when consumed in moderation. The consumption of 1–3 wine drinks/week are in line with the 2015–2020 dietary guidelines for Americans. recommending alcohol consumption of up to one alcoholic drink per day for women and up to two alcoholic drinks per day for men (one drink of wine = 150 mL) [45]. The effect of wine on obesity parameters has been inconsistent, with reports of either beneficial or adverse effects [46,47,48]. However, regardless of genetic predisposition, several epidemiological studies and clinical trials have reported a beneficial effect of wine consumption on human health with anti-obesity properties [49,50]. Wine consumption had the greatest inverse association for risk of overweight among other alcoholic beverages (hazard ratio: 0.75, 95% CI 0.68–0.84) [51]. In a large cohort (*n* = 280,183 participants), red-wine drinkers had −0.75 kg/m^2^ lower BMI compared to non-drinkers [52]. In our study, *FTO* rs9939609 wild type and heterozygotes who drank between one and three weekly drinks of wine had ≈−1.9 kg/m^2^ and 1.2 kg/m^2^ BMI reduction compared to their non-drinking wine counterparts, respectively. To date, the functional mechanisms explaining the interaction between risk of obesity, moderate wine consumption and genetic polymorphisms (including *FTO* rs9939609) have not been fully resolved due to the complexity of the factors involved. Yet, several studies suggested that the presence of resveratrol, a natural polyphenol found in grape skin with antioxidant properties, has beneficial effects on health, including body weight, BMI, adipocyte size and inflammatory cytokines reduction [53,54]. Nevertheless, the precise functional relation between wine consumption, *FTO* variants and obesity risk should be further studied.

SSB consumption has been strongly linked to obesity [55]. We found an interaction between *FTO* rs9939609, rs8050136, rs1421085 and rs1121980 polymorphism carriers and SSB on obesity risk in women. Female carriers of the rs9939609 risk allele consuming daily SSB (≥1 serving) had significantly increased obesity risk compared to non-consumers. In concordance with our results, a recent study has shown that rs9939609 A risk-allele carriers are prone to higher scores of food cravings and resistance to age-related decline in cravings [56]. Moreover, a significant association between *FTO* rs11642841 (correlated with *FTO* rs9939609) and total sugar intake was reported [57]. A potential mechanism related to the interaction of these four particular *FTO* SNPs with SSBs may be explained by their association to sugar metabolism and insulin resistance [58,59], although reports on the role of rs1421085 in glucose metabolism were inconclusive [60,61]. Our results reinforce previous findings, but more importantly, they complete a bigger picture of a potential intervention strategy to prevent the effect of SSB consumption by targeting guidelines in this genetic group predisposed to obesity, specifically in women.

We have demonstrated that eighteen studied *FTO* common SNPs are significantly associated with obesity in the Israeli population. In all eighteen *FTO* SNPs, carrying at least one risk variant allele elevated the odds of being obese (BMI ≥ 30 kg/m^2^). The *FTO* rs9939609 minor allele frequency (MAF) in our studied population was 0.48 (with similar MAF of the other SNPs), which is close to the previously reported European (0.41) and African (0.47) MAF (in line with the fact that the Israeli population originated mostly from European and African countries) [62], but it was higher than the Asian allele frequency (0.13) [11,63]. Carrying the *FTO* rs9939609 risk allele was associated with BMI increments of 0.65 kg/m^2^ (*p* = 0.015) and 1.2 kg/m^2^ (*p* = 0.002) for heterozygotes and homozygotes, respectively. These findings are in agreement with studies in Caucasian populations [11,64,65]. Our findings replicate and verify the association between *FTO* SNPs and elevated risk of obesity.

Our study has several limitations: due to the cross-sectional nature of this investigation, we cannot infer causality regarding the *FTO* SNPs and obesity, as functional variants are still unknown. Furthermore, BMI is a convenient surrogate measure for obesity, but it may be influenced by changes in bone mass and lean mass as well as adiposity. Another limitation of the study is the use of self-reported questionnaires and anthropometric measurements which may not be always accurate and to some degree rely on the weight and emotional status of the participants compared to measurement taken by professional stuff. Furthermore, self-reported weight usually tends to underestimation, especially in overweight and obese participants than those of normal weight [66]. This research studied the Israeli population, and thus, the findings may not be relevant to other populations. Our study has several strengths, including a large sample cohort, providing more reliable results with smaller margins of error. Additionally, we analyzed both several common *FTO* genetic variations and lifestyle habits. Furthermore, this is, to the best of our knowledge, the first nutrigenetics report regarding *FTO* and obesity in the Israeli population.

## 5. Conclusions

We found a significant association of eighteen common *FTO* obesity-predisposition SNPs with the risk of obesity in the Israeli population. Significant gene–environmental interaction was found for the rs9939609 *FTO*, where physical activity significantly reduces the *FTO* genetic obesity predisposition. Furthermore, consuming SSB was found to significantly elevate the *FTO* genetic obesity predisposition risk, while wine consumption was found to significantly reduce the *FTO* genetic obesity predisposition risk. Our results demonstrate actionable treatments effective in reducing elevated genetic predisposition for obesity.

## Figures and Tables

**Table 1:** Descriptive characteristics of study population by obesity status.

	All Population *n* = 1972	Obese (BMI ≥ 30) *n* = 1098	Non-Obese (BMI < 30) *n* = 874	*p*-Value
Gender (women, %)	1377 (79%)	721 (65.7%)	650 (74.4%)	<0.001
Age (mean ± SD)	55.22 ± 14.36	54.97 ± 14.54	55.53 ± 14.14	0.6
Weight (mean ± SD)	87.32 ± 19.28	98.45 ± 17.25	74.34 ± 10.69	<0.001
Hight (mean ± SD)	166.82 ±8.66	167.16 ± 8.99	166.38 ± 8.21	0.057
BMI (mean ± SD)	31.25 ± 5.82	35.11 ± 4.66	26.41 ± 2.65	<0.001
Physically active (*n*, %) *	908 (46%)	407 (37%)	501 (57.3%)	<0.001
Smoking (*n*, %) **	190 (9.6%)	97 (8.8%)	93 (10.6%)	0.279
SSB consumers (*n*, %) **	206 (10.4%)	135 (6.8%)	71 (3.6%)	0.002
Wine consumers (*n*, %) ***	385 (21.4%)	189 (18.8%)	196 (24.7%)	<0.001

SD: Standard deviation. * data for *n* = 1735 (missing data for 237 participants). ** data for *n* = 1796 (missing data for 176 participants). *** data for *n* = 1754 (missing data for 218 participants).

**Table 2:** Participants’ FTO SNPs genotype frequency and obesity risk.

		Genotype Frequency (%)				*p*-Value OR ± 95% (CI)			
SNP	Allele		Overall Population (*n* = 1972)	Obese (*n* = 1098)	Non-Obese (*n* = 874)	Dominant Model	Recessive Model	Additive Model	Codominant Model
rs9939609	T > A	TT AT AA	28.2% 46.9% 24.8%	26% 47% 27%	31% 46.8% 22.2%	0.014 1.28 (1.05–1.56)	0.014 1.3 (1.05–1.60)	0.003 1.2 (1.07–1.36)	0.003 1.46 (1.14–1.87)
rs1421085	T > C	TT TC CC	26.7% 47.8% 25.5%	25% 47.4% 27.6%	28.7% 48.4% 22.9%	0.077 1.2 (0.98–1.47)	0.016 1.29 (1.05–1.59)	0.011 1.17 (1.04–1.33)	0.01 1.38 (1.08–1.78)
rs8050136	C > A	CC CA AA	28.2% 46.6% 25.2%	26% 46.4% 27.5%	30.9% 46.8% 22.3%	0.018 1.27 (1.04–1.55)	0.008 1.33 (1.07–1.63)	0.002 1.2 (1.07–1.37)	0.002 1.47 (1.15–1.88)
rs8051591^a^	A > G	AA AG GG	27.3% 47.7% 25%	25.5% 47.1% 27.4%	29.7% 48.4% 21.9%	0.058 1.23 (0.99–1.53)	0.011 1.34 (1.07–1.68)	0.007 1.2 (1.05–1.38)	0.007 1.45 (1.1–1.9)
rs3751812 ^a^	G > T	GG GT TT	28.8% 47.6% 23.6%	27% 47.2% 25.8%	31% 48.1% 20.9%	0.074 1.21 (0.98–1.5)	0.019 1.32 (1.05–1.66)	0.012 1.19 (1.04–1.36)	0.01 1.42 (1.09–1.86)
rs9935401 ^a^	G > A	GG GA AA	27.3% 47.9% 24.8%	25.5% 47.2% 27.3%	29.7% 48.7% 21.7%	0.058 1.23 (0.99–1.53)	0.009 1.35 (1.08–1.7)	0.006 1.2 (1.06–1.38)	0.005 1.47 (1.12–1.92)
rs11075989 ^a^	C > T	CC CT TT	27.2% 48% 24.8%	25.2% 47.8% 27.1%	29.8% 48.3% 21.9%	0.033 1.27 (1.02–1.57)	0.016 1.32 (1.05–1.66)	0.006 1.2 (1.06–1.38)	0.006 1.47 (1.12–1.92)
rs9923233 ^a^	G > C	GG GC CC	27.4% 47.7% 24.9%	25.4% 47.4% 27.2%	29.9% 48.1% 21.9%	0.038 1.26 (1.01–1.56)	0.014 1.33 (1.06–1.66)	0.006 1.2 (1.06–1.38)	0.006 1.46 (1.12–1.92)
rs9936385 ^a^	T > C	TT TC CC	27.4% 47.8% 24.8%	25.4% 47.4% 27.2%	29.9% 48.3% 21.8%	0.038 1.26 (1.01–1.56)	0.012 1.34 (1.07–1.68)	0.005 1.2 (1.06–1.39)	0.005 1.47 (1.13–1.93)
rs17817964 ^a^	C > T	CC CT TT	28.8% 47.3% 23.8%	27.1% 46.8% 26.1%	31% 48% 21%	0.086 1.2 (0.97–1.49)	0.016 1.33 (1.06–1.67)	0.013 1.19 (1.04–1.36)	0.011 1.42 (1.09–1.86)
rs8043757 ^a^	A > T	AA AT TT	27.3% 47.7% 24.9%	25.4% 47.2% 27.4%	29.8% 48.4% 21.8%	0.045 1.25 (1–1.55)	0.009 1.35 (1.08–1.7)	0.005 1.2 (1.06–1.39)	0.005 1.47 (1.13–1.93)
rs1121980 ^a^	G > A	GG GA AA	21.1% 48.5% 28.4%	21.3% 48.2% 30.5%	25.4% 48.8% 25.8%	0.06 1.24 (0.99–1.56)	0.037 1.26 (1.01–1.56)	0.015 1.18 (1.03–1.35)	0.016 1.4 (1.07–1.83)
rs17817449 ^a^	T > G	TT TG GG	27.6% 47.5% 24.9%	25.7% 47% 27.3%	30.1% 48.1% 21.8%	0.5 1.24 (1–1.54)	0.011 1.34 (1.07–1.68)	0.006 1.2 (1.06–1.38)	0.005 1.46 (1.12–1.92)
rs62033400 ^a^	A > G	AA AG GG	28.8% 47.6% 23.6%	27% 47% 26%	31% 48.4% 20.6%	0.077 1.21 (0.98–1.5)	0.011 1.35 (1.07–1.7)	0.009 1.2 (1.05–1.37)	0.007 1.45 (1.1–1.9)
rs7202116 ^a^	A > G	AA AG GG	27.3% 47.7% 25%	25.3% 47.5% 27.2%	29.9% 47.9% 22.2%	0.033 1.26 (1.02–1.57)	0.02 1.3 (1.04–1.64)	0.007 1.2 (1.05–1.38)	0.007 1.45 (1.1–1.9)
rs7193144 ^a^	T > C	TT TC CC	27.3% 47.8% 24.9%	25.4% 47.3% 27.3%	29.8% 48.4% 21.8%	0.045 1.25 (1–1.55)	0.011 1.34 (1.07–1.69)	0.006 1.2 (1.05–1.38)	0.005 1.47 (1.12–1.92)
rs11075990 ^a^	A > G	AA AG GG	27.2% 48% 24.8%	25.2% 47.8% 27.1%	29.8% 48.3% 21.9%	0.033 1.27 (1.19–1.57)	0.016 1.32 (1.53–1.66)	0.006 1.2 (1.06–1.38)	0.006 1.46 (1.12–1.92)
Rs6499640 ^a^	G > A	GG AG AA	17.3% 47.9% 34.8%	19.2% 45.8% 35%	14.8% 50.5% 34.6%	0.022 0.74 (0.57–0.96)	0.842 1.02 (0.83–1.25)	0.269 0.93 (0.81–1.06)	0.09 0.78 (0.58–1.05)
Rs13333228 ^b^	T > C	TT TC CC	5.5% 30.6% 64%	4.9% 29% 66.1%	6.2% 32.5% 61.3%	0.217 1.3 (0.86–1.99)	0.034 1.24 (1.2–1.52)	0.028 1.2 (1.02–1.41)	0.129 1.4 (0.9–2.14)
rs1558902 ^b^	T > A	TT TA AA	25.9% 48.5% 25.6%	24.5% 47.7% 27.8%	27.7% 49.5% 22.8%	0.166 1.17 (0.94–1.46)	0.019 1.3 (1.04–1.63)	0.023 1.17 (1.02–1.34)	0.022 1.37 (1.05–1.8)
rs9302652 ^b^	C > T	CC CT TT	9.8% 45.7% 44.5%	9.4% 45.5% 45.1%	10.3% 45.9% 43.8%	0.502 1.12 (0.81–1.54)	0.384 1.3 (0.99–1)	0.477 1.06 (0.91–1.22)	0.456 1.14 (0.81–1.6)

Logistic regression was adjusted for age and gender. OR: odds ratio; CI: confidence interval. ^a^: total *n* = 1686; obese *n* = 938; non-obese *n* = 748. ^b^: total *n* = 1685; obese *n* = 938; non-obese *n* = 747.

**Table 3:** FTO rs9939609 SNP population characteristics.

	Total (*n* = 1972)	TT (*n* = 506)	TA (*n* = 843)	AA (*n* = 447)	*p*-Value
Age	55.22 ± 14.36	54.1 ± 14.5	55.5 ± 14	55.9 ± 14.8	0.1
BMI	31.25 ± 5.82	30.66 ± 5.74	31.3 ± 5.68	31.86 ± 5.74	0.004
Hight	166.82 ± 8.66	166.84 ± 8.33	166.6 ± 8.82	167.20 ± 8.76	0.455
Weight	87.32 ± 19.28	85.72 ± 19.23	87.18 ± 18.79	89.4 ± 20.11	0.008
Obese (BMI > 30), *n*, (%)	1098 (55.7%)	286 (51.3%)	516 (55.8%)	296 (60.4%)	0.013
Physically active *n*, (%)	908 (46%)	255 (50.4%)	413 (49%)	240 (53.7%)	0.38
SSB consuming *n*, (%)	206 (10.5%)	70 (13.8%)	88 (10.4%)	48 (10.7%)	0.16
Wine consuming *n*, (%)	385 (19.5%)	109 (22.1%)	175 (21.4%)	101 (22.9%)	0.98

Data are presented as mean ± SD for continuous variables or as *n*; number of samples.

**Table 4:** Physical activity effect on *FTO* rs9939609 obesity risk.

	Physically Inactive			Physically Active		
	β	Obesity (BMI > 30) OR ± CI	*p*-Value	β	Obesity (BMI > 30) OR ± CI	*p*-Value
rs9939609 AA + TA vs. TT	0.336	1.4 (1–1.93)	0.04	0.1	1.1 (0.82–0.86)	0.4
rs9939609 AA vs. TA + TT	0.53	1.78 (1.23–2.57)	0.002	0.13	1.14 (0.84–0.86)	0.4
rs9939609 AA vs. TT	0.174	1.99 (1.3–3.05)	0.002	0.174	1.19 (0.83–1.7)	0.34

Logistic regression was adjusted for age and gender. OR: odds ratio; CI: confidence interval.

**Table 5:** Physical inactivity and wine consumption effect on *FTO* rs9939609 obesity risk.

	Physical Inactivity			Wine Consumption		
	β	OR ± CI	*p*-Value	β	OR ± CI	*p*-Value
rs9939609 TT	0.826	2.29 (1.58–3.3)	<0.001	−0.582	0.56 (0.36–0.87)	0.009
rs9939609 TA	0.934	2.54 (1.91–3.39)	<0.001	−0.554	0.58 (0.4–0.8)	<0.001
rs9939609 AA + TA	1.048	2.86 (2.25–3.6)	<0.001	−0.386	0.68 (0.52–0.89)	<0.005
rs9939609 AA	1.326	3.77 (2.47–5.75)	<0.001	−0.085	0.92 (0.58–1.45)	0.715

Logistic regression was adjusted for age and gender; OR: odds ratio CI: confidence interval; Regular wine consumption: 1–3 weekly drinks.

**Table 6:** Interaction between SSBs consumption and *FTO* SNPs on obesity risk (BMI > 30) in women.

SNP *n* = 1746	Obesity Risk (Interaction with SSB) OR ± CI	*p*-Value
rs9939609	2.23 ± (1.33–3.76)	0.002
rs8050136	2.23 ± (1.33–3.75)	0.002
rs1421085	2.3 ± (1.37–3.85)	0.002
rs1121980	1.9 ± (1.12–3.31)	0.019

Logistic regression adjusted for age. Assuming dominant model.

## Data Availability

Not applicable.

## Supplementary Materials

The following supporting information can be downloaded at: https://www.mdpi.com/article/10.3390/nu14194202/s1, Table S1: FTO SNP’s interaction with physical activity.
