# Supplementary material for: FTO Common Obesity SNPs Interact with Actionable Environmental Factors: Physical Activity, Sugar-Sweetened Beverages and Wine Consumption
Source: Nutrients. 2022 Oct 9;14(19):4202. doi: 10.3390/nu14194202 (PMC9572787; doi:10.3390/nu14194202)
Supplement: Supplementary file 1 [file nutrients-14-04202-s001.zip › nutrients-1922968-supplementary.pdf]

Table S1: *FTO* SNPs interaction with physical activity

SNP (n=1735)	Dominant mode		Recessive model	
	OR ± 95% CI	P-value	OR ± 95% CI	P-value
Rs9939609	2.52 (2.04-3.12)	<0.001	2.94(2.08-4.16)	<0.001
Rs8050136	2.53 (2.05-3.13)	<0.001	2.9(2.07-4.07)	<0.001
Rs8051591	2.23 (1.78-2.8)	<0.001	2.8(1.9-4)	<0.001
Rs3751812	2.22 (1.76-2.8)	<0.001	2.8(1.9-41)	<0.001
Rs9935401	2.23 (1.78-2.8)	<0.001	2.76(1.9-4)	<0.001
Rs11075989	2.24 (1.78-2.8)	<0.001	2.82(1.9-4.1)	<0.001
Rs9923233	2.23 (1.77-2.8)	<0.001	2.74(1.9-3.97)	<0.001
Rs9936385	2.22 (1.77-2.8)	<0.001	2.82(1.9-4.1)	<0.001
Rs17817964	2.2 (1.75-2.76)	<0.001	2.91(1.9-4.28)	<0.001
Rs13333228	2.3 (1.85-2.85)	<0.001	2.08(1.65-2.63)	<0.001
Rs9302652	2.22 (1.79-2.76)	<0.001	1.69(1.3-2.2)	<0.001

Rs8043757	2.23 (1.78-2.8)	<0.001	2.76(1.9-4)	<0.001
Rs1121980	2.32 (1.85-2.9)	<0.001	2.64(1.87-3.73)	<0.001
Rs1558902	2.23 (1.77-2.79)	<0.001	2.57(1.8-3.66)	<0.001
Rs17817449	2.23 (1.78-2.81)	<0.001	2.76(1.9-4)	<0.001
Rs7202116	2.22 (1.77-2.79)	<0.001	2.74(1.89-3.97)	<0.001
Rs62033400	2.22 (1.77-2.8)	<0.001	2.82(1.93-4.14)	<0.001
Rs7193144	2.23 (1.78-2.8)	<0.001	2.76(1.9-4)	<0.001
Rs11075990	2.23 (1.78-2.8)	<0.001	2.82(1.94-4.1)	<0.001
Rs6499640	1.75(1.4-2.18)	<0.001	1.64(1.23-2.18)	<0.001
Rs1421085	2.5 (2.03-3.1)	<0.001	2.6 (1.88-3.6)	<0.001

OR: Odds ratio
CI: confidence interval
Adjusted to age and gender
